# The contrasting role of technology as both supportive and hindering in the everyday lives of people with mild cognitive deficits: a focus group study

**DOI:** 10.1186/s12877-018-0879-z

**Published:** 2018-08-17

**Authors:** Eva Lindqvist, Annika PerssonVasiliou, Amy S. Hwang, Alex Mihailidis, Arlene Astelle, Andrew Sixsmith, Louise Nygård

**Affiliations:** 1 Department of Neurobiology, Care Sciences and Society (NVS), Division of Occupational Therapy, Karolinska Institutet, Fack 23 200, SE-141 83 Huddinge, Sweden; 2 University of Toronto and Toronto Rehab Institute-UHN, Toronto, Canada; 3 University of Sheffield, Sheffield, UK; 4 Simon Fraser University, Vancouver, Canada

**Keywords:** Older adults, Mild cognitive impairment, Dementia, Technology, Environment, Support

## Abstract

**Background:**

It is well known that people with mild cognitive deficits face challenges when performing complex everyday activities, and that the use of technology has become increasingly interwoven with everyday activities. However, less is known of *how* technology might be involved, either as a support or hindrance, in different areas of everyday life and of the environments where challenges appear. The aim of this study was to investigate the areas of concern where persons with cognitive deficits meet challenges in everyday life, in what environments these challenges appear and how technology might be involved as part of the challenge and/or the solution to the challenge.

**Methods:**

Data were gathered through four focus group interviews with participants that live with cognitive deficits or cohabit with a person with cognitive deficits, plus health professionals and researchers in the field. Data were transcribed, coded and categorized, and finally synthesized to trace out the involvement of technology.

**Results:**

Five areas of concern in everyday life were identified as offering challenges to persons with cognitive deficits: A) Managing personal finances, B) Getting around, C) Meeting family and friends, D) Engaging with culture and media and, E) Doing everyday chores. Findings showed that the involvement of technology in everyday activities was often contrastive. It could be hindering and evoke stress, or it could bring about feelings of control; that is, being a part of the solution. The involvement of technology was especially obvious in challenges linked to Managing personal finances, which is a crucial necessity in many everyday activities. In contrast, technology was least obviously involved in the area Socializing with family and friends.

**Conclusions:**

The findings imply that technology used for orientation and managing finances, often used outside home, would benefit from being further developed in order to be more supportive; i.e. accessible and usable. To make a positive change for many people, the ideas of inclusive design fit well for this purpose and would contribute to an age-friendly society.

**Electronic supplementary material:**

The online version of this article (10.1186/s12877-018-0879-z) contains supplementary material, which is available to authorized users.

## Background

This study is part of the Ambient Assistive Living Technologies for Wellness, Engagement, and Long Life project (AAL-WELL) [1], aiming at exploring how innovative technologies could support daily activities among older people with mild cognitive deficits. It is well known that people with mild cognitive deficits face challenges when performing complex everyday activities, both inside and outside the home, for example, when managing finances [2, 3], remembering appointments [3, 4], or reading books [3]. Yet, the role of technology in everyday activities is not equally well explored when it comes to users with mild cognitive deficits. Research has however shown that these people have to handle more explicit obstacles related to maintaining activities that include everyday technology than healthy older adults, and that they take on different approaches for that, for examples downsizing by ceasing to use technology or downsizing activities, which could include incorporation of new technology for support [5]. Increasing the knowledge of the role of technology is of great need as mild cognitive deficits are common in the ageing population. These deficits can be due to a minor stroke [6], early stage dementia [7], or mild cognitive impairment (MCI), sometimes described as a condition between normal aging and early Alzheimer’s disease [8], although a large proportion of those with MCI do not develop dementia [9]. Hereafter, the abbreviation CD will be used as an overarching term for mild cognitive deficits due to these conditions.

This study has its focus on everyday technologies, that is; electronic, technical and mechanical artefacts that exist in people’s lives at home and in the community [10], with the focus on electronic everyday technology, from now on referred to as technology. Even if technology has been a natural part in most people’s lives for many decades – for example, washing machines, television – it is obvious that it has become more integrated in everyday activities both in domestic and public life today. It has also become more expected in society, that everyone can manage technology competently and independently, both at home, e.g., remote controls, microwave ovens and personal computers, and in public space, e.g., ticket vending machines or parking meters. The challenges encountered by persons with CD when using technologies have been well described (see for example [5, 11, 12]) although not in relation to the role of technology or to the environments where challenges occur. It is also recognized that to maintain engagement and independence in activities in everyday life, persons with CD have to tackle situations linked to technology, and how these situations are met have shown to be of crucial value for retaining the ability to use technology [13–15]**.** At the same time, it is also important to keep in mind the positive outcomes from using the technology, since it can be regarded as a facilitator in everyday life for persons with CD [5, 16], and avoiding technology is neither possible nor desired. This suggests that more in-depth inquiry is needed into the interactions between challenges in everyday activities, technology’s role and how persons with CD try to meet and find solutions to these challenges.

There are great expectations in society that technology will provide solutions to a variety of challenges. The hopes are particularly high that so called ambient assisted living (AAL) technologies and services (i.e. technologies that can sense a person’s activity or behavior and provide tailored support as needed [17]) will increase autonomy, self-confidence and mobility, and, further, prevent social isolation, enhance security and continued living in an individual’s preferred environment [17]. Using technology as support is also a common answer to the question how to afford the expected costs linked to an aging population [18], and many local and international initiatives have been launched to encourage research and development in this field [17]. However, the everyday priorities as expressed by persons with CD, and how these priorities link to technology use, have commonly received less attention. To guide the development of relevant technological support, be it ambient assisted living technologies and services or common everyday technologies, a literature review was first conducted in the AAL-WELL project [19] to identify important but challenging daily activities that persons with CD wanted to continue mastering and why. Although it is well known that the physical environment, e.g. the home or public space, is of major importance for activities of daily living, and also for competent use of technology [20], this literature review [19] pointed out that the empirical information concerning the physical environment involved when persons with CD do their challenging everyday activities is sparse. The findings described reasons for why the challenging activities were desired, and relationships of dependence between activities were found; some activities were prerequisites for other activities. The most difficult activities seemed to hinder outdoor life, but beside that, environmental aspects did not come to the fore although evidently of importance. This led us to continue exploring this topic in the present focus group study.

Having insights into the types of activities that persons with CD prioritize and where they take place, the nature of the challenges that occur and how challenges are met is paramount for developing any support, including technologies for cognitive support that can lead to positive changes for these individuals. The importance of considering environmental factors when designing and developing technological support for people with various needs has been stated previously [21–23]. The Person-Environment-Occupation Model [24] highlights the strong relationship between a person’s activities and the environment, also pointing out how the transactional dynamics between a person’s roles, self-concept, abilities, culture and background, the environment – the technology being part of the environment - and the clusters of activities that meet intrinsic needs, together form the outcome of activity performance. This model also supports the importance of placing the activity in focus when examining the relationship between persons and their environments, a feature that is critical for designing appropriate technology [25]. Based on the identified knowledge gap, this study set out to investigate the areas of concern where persons with CD meet challenges in everyday life, in what environments these challenges appear and how technology might be involved as part of the challenge and/or solution to challenges. Our intention is to provide knowledge to facilitate the development of support, particularly the utilization and creation of technological solutions that are relevant to the expected users’ priorities, taking the environmental aspects as well as the kind of solutions people with CD might have chosen into consideration.

## Methods

### Design

This study is a qualitative study in which data were gathered through focus group interviews [26, 27] conducted with the purpose of deepening the understanding and widening the view of the topic; that is the areas of concern where persons with CD meet challenges in everyday life, in what environments these challenges appear and how technology might be involved as part of the challenges and/or solution to challenges [27]. Using focus groups can provide valuable information for developing new solutions [27]. We chose to invite a variety of participants as informants to the focus group interviews. The assumption was that having different backgrounds, prior understandings and perspectives among the participants would enrich the data gathered. Ethical approval was obtained from the ethical board in Stockholm; protocol 2013/833–31/3.

### Recruitment and participants

In order to gather as extensive data as possible, three types of stakeholders with different perspectives were recruited with a convenient sampling approach:

1. *Health professionals at memory investigation clinics,* (*n* = 4; Licensed Occupational Therapists [*n* = 3], Licensed Psychologist [*n* = 1]). These health professionals were experienced in conducting clinical interviews with persons with CD about their priorities as well as difficulties in everyday lives, both at the clinic and during home visits. Among their clients were people who might not typically be interested in being involved in research and whose views would therefore not ordinarily be available to researchers. This group was a ‘naturally occurring’ group, that is, colleagues at the same clinic, which can be beneficial because they can relate to each other’s comments, according to Kitzinger [26].
2. *Researchers in the area of MCI/early stage dementia and public environment/technology (n = 5)*. These researchers had a background in occupational therapy and had conducted extensive interviews with persons with CD and their significant others in a variety of studies focusing on understanding the person’s situation, but not on assessing their abilities or doing interventions, unlike the health professionals’ purposes. This was also a ‘naturally occurring’ group.
3. *Members of volunteer health organizations.* For the recruitment of the participants from volunteer health organizations the term “mild cognitive deficits” was defined in an everyday language by the following words: *A person who manages relatively well in daily life, but due to cognitive deficits can use a little support now and then from others or from technology; a person who does things a bit more slowly or is in need of paying more attention to detail than others.* The participants had experiences of living with the consequences of cognitive deficits in their everyday lives derived from having mild cognitive deficits themselves (*n* = 5), or from cohabiting with someone with mild cognitive deficits (n = 5). They were divided into two mixed groups, both including persons with CD and spouses of persons with CD as they all volunteered to participate as equal members of volunteer health organizations; i.e. the voices of the persons with CD were given equal weight as the voices of their spouses. The purpose of the mixed groups was to get different perspectives and the reflections on the perceived challenges that persons with CD meet in everyday life and thereby get a more comprehensive understanding of them. In one case, a couple was participating in a focus group; in all other cases there were no close relationships between the participants. The participants with CD and their spouses were considered the most important ones in terms of giving their perspective on data from the other two focus groups. Hence, these focus groups were conducted after the other two groups, in order to allow more in-depth discussions on topics that had emerged (Table 1).

**Table 1:** Demographics of participants in focus groups

Focus group 1: Professionals at a memory investigation clinic					
Sex	35–50 years	51–60 years	61–70 years	Years in the field	Profession
F		X		3	Leg. Occupational Therapist (OT)
F	X			11	Leg. OT
F			X	13	Leg. OT
M	X			2	Leg. Psychologist
Focus group 2: Researchers in MCI/Dementia, technology and public space					
Sex	35–50 years	51–60 years	61–70 years	Years in the field	Profession
F	X			16	Doctoral student, Leg. OT
F	X			20	Doctoral student, Leg. OT
F	X			20	Ph. D./Lecturer, Leg. OT
F	X			14	Ph. D./Lecturer, Leg. OT
F	X			20	Doctoral student, Leg. OT
Focus group 3 and 4: Members of volunteer Health Organizations					
Sex	35–50 years	51–60 years	61–70 years	Years of member ship	Member in:
F			X	2	Alzheimer association and Dementia association
F			X	1	Dementia association
M			X	1	Dementia association
F		X		0	Spouse
F			X	4	Alzheimer association and Dementia association
F			X	2	Alzheimer association and Dementia association
F		X		13	Brain Injury association
F			X	4	Alzheimer association and Dementia association
F			X	4	Alzheimer association and Dementia association
F			X	3	Dementia association

Participants in the first two groups had already been identified by the research team and were contacted directly for the interviews. With this approach, it was made certain that participants had an interest in and insight into the matter. They were considered able to be “good informants” [26], that is, they were able to reflect on a range of issues relating to the population of interest; that is people experiencing mild cognitive deficits. For recruitment of members of volunteer health organizations, three organizations whose aim was to support people with cognitive deficits were contacted. Their central administrations then either sent a request by e-mail to their members about the study and/or recommended that the researchers visit their social gatherings. Each local organization was then contacted, and they approved and invited the researchers to introduce the study at social gatherings. Interested members contacted the researchers via e-mail, telephone, or in person (at the social gathering) to approve participation. In total, one participant was recruited via the e-mail request, and nine participants were recruited via the social gatherings.

### Data collection

The first focus group (health professionals) was conducted at the memory investigation clinic where the participants worked. The session lasted for 90 min. The second focus group (researchers) was conducted at the university just outside Stockholm, and lasted for 2 h. The third and fourth focus groups (members of health organizations) gathered at a geriatric clinic in a central part of Stockholm, and each of the group sessions lasted for 2 h. The clinic was chosen for its central location, as recommended by Murphy [27]. At every focus group session, there were two persons from the research team present. One was a facilitator (EL) who led the discussion based on the topics in the interview guide [26]. The other one (APV) took notes in order to keep a record of whether all topics were covered and if topics discussed in previous groups were mentioned. Refreshments were served and there were opportunities for presentations and some small talk before the discussions, with the purpose of creating a non-threatening atmosphere [27]. In the focus group introduction, the facilitator encouraged the participants to actively take part in the discussions and to ask each other questions during the session. It was also underscored that consensus was not of importance, but that all opinions were of interest. An interview guide (See the Additional file 1 Topics guide) was prepared and used as a flexible support with the purpose of ensuring that all main topics had been covered by the end of each session [27]. The facilitator led and followed the discussion and asked clarifying questions when needed.

The specific questions in the guide were only asked if the participants did not bring up the subject in the discussion. The questions focused on experienced or observed challenges in everyday life due to cognitive deficits, on challenging activities that were important to master, as well as on environmental aspects related to these challenges and priorities. In case participants only described observed challenges among persons with CD, the facilitator tried to encourage descriptions of how the persons with CD in those particular cases had expressed their experiences of these challenges, as one purpose of the focus group discussions was to capture possible priorities of persons with CD. In the last part of each focus group, questions were raised about issues that had been identified in the previous focus groups or in the literature, and that had not been mentioned in the current focus group. Examples of questions were: *The previous group mentioned that xx (xx representing an issue from an earlier focus group). Have you any experiences of that? What do you think of xx, is it a problem? How important do you think xx in general is to persons with CD?* In this way, it was possible to get a variation of perspectives and to enrich the previous data. A final question before wrapping up was posed about whether something was important to add. The interviews were audio-recorded in order to capture the conversation fully.

### Data analysis

The focus group discussions were transcribed verbatim and entered into NVivo 10 (QSR International). First, the analysis focused on identifying the activities that, according to focus group participants, were perceived as challenging and important by persons with CD. It was considered fruitful to view the different groups’ perspectives as complementary to each other instead of using their different contributions for comparisons, which led to all data being treated as one set [28]. All data that could be of interest for the aim of the study were labeled with concrete and explanatory codes. Examples of codes are: “risk of losing the credit card at the ATM”, “difficult to handle stress related to trips abroad”. These codes were inductively categorized into five *areas of concern* that emerged from the data [29]. Discussions that were not relevant to the aim, such as challenges related to being a spouse of a person with CD, were not coded regardless of their importance to the individual.

In a next step, each *area of concern* was searched through in order to find the various environmental factors related to challenges that had been reported by the participants. In order to more thoroughly analyze the challenges and the involvement of technology in these challenges, the extracts of data where challenges and their relation to technology came to the fore were categorized for each challenge in a matrix with given codes based upon the aim. These data were examined and questions were posed in order to find similarities, differences and patterns by continuous comparisons within and across areas of concerns.

Finally, the findings were synthesized into written text, using the areas of concern as category headings, describing the challenges, the environments, the role of technology, and the solutions found, as well as the eventual consequences of all these for each area of concern. These are summarized in separate tables within each of the five areas of concern. The order of the areas of concern in the presentations was based upon the degree of urgency for solutions as interpreted from the data. To further clarify the involvement of technology as part of the problem and/or the solution, a summary is presented in the end of the result section.

## Results

In the findings, the challenges and how they were met according to the participants is presented in the five inductively created areas of concern; A) Managing personal finances, B) Getting around, C) Meeting family and friends, D) Engaging with culture and media, and (E) Doing everyday chores. In each area, the synthesised text under each area’s heading presents where the challenges mostly appeared, with a specific focus on how the technology was involved when meeting the challenges. Also, the approaches used by persons with CD when meeting the challenges are described. Tables 2, 3, 4, 5 and 6 provide an overview of the results for each area of concern.

A) Managing personal finances: Technology adds stress to an already stressful activity but can also bring control

**Table 2:** Reported challenges, environments, technology involvement and approaches related to Managing personal finances

Challenge	Physical environment	Technology - part of problem	Technology - part of solution	Approach to meet the challenge
Manage Internet banking	At home	Bank website		Use paper forms again
Make safe online purchase	At home	On-line shopping website		
Keep track whether bills are paid	At home		Internet bank on online computer	Check paid invoices at bank account online
Pay bills on time	At home		Mobile phone/PDA	Insert reminders in mobile phone
Avoid being fooled by telemarketing sales-persons and door-to-door salespersons/imposters	At home	(Telephone)		
Withdraw money from ATM	Public space	ATM		Use ATM when less crowded
Manage ticket vending machines	Public space	Vending machine		
Manage payment	E.g., grocery store	Payment terminal Automated checkout station		Choose familiar cashier, pay with notes instead of coins, use invoice instead of cash
Keep track on expenses	Grocery store		Self scanning	Check expenses on self scanning display when shopping

**Table 3:** Reported challenges, environments, technology involvement and approaches related to Getting around

Challenge	Physical environment	Technology - part of problem	Technology - part of solution	Approach to meet the challenge
Leave home safely	At home	Stove/coffee machine	Mobile/smart phone, PDA	Set reminders to allow sufficient time for leave safely
Find one’s way	Outside home, various areas		Mobile/smart phone (with GPS)	Use app-lications in smart phones for GPS, choose known areas, ask someone, take a taxi, have a companion or companion dog, visit places when less crowded
Remain together with companion	Outside home, various areas		Mobile/smart phone, PDA	Call each other
Find one’s way in public transport	Public transport		Website of own on line computer	Print the planned trip ahead via website
Use GPS for orientation	Outside home, various areas	GPS app	Mobile phone/PDA

**Table 4:** Reported challenges, environments, technology involvement and approaches related to Meeting family and friends

Challenge	Physical environment	Technology - part of problem	Technology - part of solution	Approach to meet the challenge
Attend to birthdays	At home		Mobile phone/PDA	Use paper calendar and reminders in mobile phone/PDA
Be on time for appointments	At home/at family and friends’		Mobile phone/PDA	Use paper calendar and reminders in mobile phone/PDA
Engage in conversations in the social setting at hand	At family and friends’, associations			Withdraw in conversations, avoid social gatherings

**Table 5:** Reported challenges, environments, technology involvement and approaches related to Engaging with culture and media

Challenge	Physical environment	Technology- part of problem	Technology- part of solution	Approach to meet the challenge
Turn on media for watching a program	At home	TV, DVD, remote control		Stop using
Enjoy or take in the content in e.g. film or newspaper	At home			Choose less complex books, re-read books, solve crosswords intended for children
Handle media items	At home/at family and friends’	Smart phones, tablets

**Table 6:** Reported challenges, environments, technology involvement and approaches related to Doing everyday chores

Challenge	Physical environment	Technology- part of problem	Technology- part of solution	Approach to meet the challenge
Turn off kitchen equipment after use	At home/kitchen	Stove/coffee machine		
Follow recipes and instructions	At home/kitchen	E.g. Coffee machine		Cook known meals, write own instructions
Adhere to routines; e.g. Take medicine, or eat regularly	At home		Mobile phone/PDA	Insert reminders in mobile phone
Handling online booking system for common laundry room	At home	The landlord’s website		Get help from relative for booking
Buy the planned items in shop	In grocery store		Mobile phone	Call relative with mobile phone for recalling shopping list

Managing personal finances, an activity performed regularly, stood out from other activities due to its profound influence on the everyday lives of persons with CD. Challenges related to managing personal finances were primarily met at home, but also in public space and in shops. Many of the challenges were linked to online activities.

Worries, stress or insecurity were particularly reported when paying bills with Internet banking services. Challenges linked to using the computer for online purchases were also described, mostly related to finding and making use of online information and instructions when making the transactions, inputting the right digits and finding the right item. Because of these challenges, some people reported returning to using paper forms again instead of Internet banking to ensure accuracy. However, some individuals with CD preferred the Internet bank as there were certain control functions in the system, such as viewing the transaction information afterwards, and because it also allowed a direct debit system. There were also challenges related to remembering to pay the household bills and keeping track of whether they had been paid, which could lead to repeated checking of payments. To avoid that, inserting automatic reminders in their smartphones and PDAs (Personal Digital Assistants), was one solution people used to handle the problem. Another solution participants described was to develop structures for how to store bills before payment to maintain control. One challenge related to managing finances at home was the risk of being fooled by telemarketing salespersons or by door-to-door salespersons. These salespersons - and there were even experiences of imposters - had to be handled, but no solutions were mentioned in this area, neither with nor without the support of technology.

Outside of the home, challenges in managing finances were linked to online technology in terms of withdrawing money from ATMs or buying tickets for public transportation in ticket vending machines, which both demand the user to understand and input information. Feelings of exposure and being an “easy victim” for criminals at the ATM were common. Withdrawing money from an ATM when there were fewer people around was one solution used to avoid such stressful situations.

Most of the focus in the discussions about managing finances was on money transactions in stores. There were perceptions of vulnerability, exposure and fear of being taken advantage of by dishonest people while paying for things. Challenges in, for example, handing over the right amount of money or remembering the pin code to the credit card, were related to stress and worries. The awareness of a need to be cautious was evident, and one participant (a researcher) cited a person with CD that she had met*: “ They (other people) can tell that I don’t behave like others, that dementia is sort of imprinted in my eyes’...”.*

Automated checkout stations in stores were often avoided and using technology as support in these cases was very rare. One participant (a person with CD), however, stated that using self-scanning when shopping helped her to keep track of expenses and to avoid the stress at the cashier. Overall, the solutions were often related to avoiding challenging situations, for example, to only pay with notes and never with coins in order to conceal difficulties in counting, to choose a familiar cashier to avoid being fooled or to ask for an invoice in the local store to completely avoid using money. If possible, some also brought a companion as support when shopping.

B) Getting around: Technology brings hope for reducing risk and fear of losing orientation

Being able to get around to places came to the fore as a prerequisite for having access to most activities, including those presented in the other areas of concern. The challenges to getting around were mostly identified outside home, although some challenges also occurred at home. Leaving home in a safe and controlled manner was described as crucial and difficult for persons with CD, and common challenges were, for example, to find needed belongings and to check that appliances, especially the stove, were turned off and all doors were locked before leaving home. The perceived risk of not leaving the home safely was related to fears of break-ins or fire accidents. Also, the risk of being delayed or even missing appointments if one could not find the key, and of leaving the home unlocked was a cause of worry, which is shown in this conversation:

A participant with CD said: * I have a balcony, and I have the door locked. The other day, when I was about to leave for my grandchild’s first birthday party, I couldn’t find the key.*

Another participant with CD asked: *To the balcony door?*

First participant replied: *To the balcony door, yes. And I looked for it, and looked for it. But then I had to leave, so I left. Otherwise, I would have missed the whole birthday party. Then I searched through (the home) when I came back, and I hadn’t had a break-in anyway. You see, I have had two break-ins in the apartment before* (someone: *oh dear!*) *so I was so worried.*

Reported solutions were described both without and with support from technology; for example to attach valuables to strings in one’s purse in order not to lose them and to set reminders about time to leave in the smartphone in order to allow sufficient time for preparations.

Getting around outside home was viewed on the one hand as crucial for maintaining valuable activities, and related to increased confidence and self-esteem, and on the other hand closely related to feelings of fear. One common challenge was to find one’s way when going for a stroll in the neighborhood, woods, and fields. Getting lost was viewed as very possible and described as a very traumatic experience. It influenced people’s self-image and confidence negatively, which was expressed by one participant with CD as *“One does not trust oneself any longer”*. Another participant with CD said: *But ... I get... because it happened to me once that I lost my way, but it is the only time. Once when I went into... I live in [village]. I went to [the small town] where I’ve been lots of times, and suddenly, I didn’t know where I was. It was so awful. I walked around. I remember that, that I walked around, looked and didn’t understand a thing, I didn’t understand. I think I went into some stores and asked. And I still don’t know how I got home, actually. So it was really... that’s why I am a bit afraid when I go out.*

It was not unusual that persons with CD had stopped going for walks in the vicinity because of the fear of getting lost, and this was described as a loss since daily walks were highly valued as recreation and for social values. Many solutions in order to continue these walks without support from any technology were described, for example, choosing known areas, asking someone in the street for help. Having the spouse or a friend as a companion was one common solution, but some persons with CD instead emphasized the importance of independence. Another more unusual solution was to have a trained companion dog for the purpose of orientation, which gave the owner hope to continue being able to get around outside independently.

Not surprisingly, the mobile or smart phone was viewed as very important for orientation. It was used for instructions from family members on how to find one’s way, and the family also felt assured, knowing that they could reach each other. Since retrieving information from ordinary maps was perceived as difficult, some used applications for GPS orientation in order to get information about their position and directions to their planned targets. The participants had met contradictory feelings about the GPS in persons with CD. On the one hand, a need for the GPS service was expressed and the GPS was described as being “*great”* by some persons with CD, and on the other hand, there was a hesitation whether they would make use of it in a stressful situation. One said: *“I won’t recall how I will know which buttons to push and know precisely...” (Person with CD).* It was difficult to remember or understand how to provide correct information required to receive support from the GPS, as well as how to use the information given from the GPS about directions, especially when difficulties in distinguishing right from left were present. The efforts that were made to make it work, despite all difficulties, indicated that this service still lent hope to the participants that it would be possible to get around outside.

Traveling by public transportation was a challenging activity and a prerequisite for visiting valued places, such as sports clubs. Apart from the earlier mentioned difficulties related to using vending machines for payments, the challenges were linked to the need to attend to important details in the environment, both in the streets and underground, especially in traffic in order to orientate. The difficulties in orientation could be seen in losing orientation in the subway or not finding one’s parked car.

Traveling was overall challenging to persons with CD according to the participants. At vacation spots new challenges occurred, as neither the hotel room nor the surroundings were familiar, which increased the risk of getting lost, and getting lost could convey feelings of panic. This was described by one participant with CD: *We were traveling somewhere (by train) and I don’t remember where we were...and it was in the evening. When we arrived at a station, I thought we should get off there, because we had stood up and were standing there. But in fact we weren’t to get off there, but I thought so and I am always in a hurry, so I took my bag and got off there, and then I saw the others (friends) standing there (in the train), staring at me. And I was standing alone on the platform. I understood that I shouldn’t have gotten off, and I tried to get on the train again, but it left the platform... the panic I felt there on the railway station... (traveling with friends in Italy).*

The cell phone was described as a necessity if they lost their companions when traveling and some could not imagine how to manage without it. Since traveling had become relatively demanding, it was described as not always worth the effort, and some persons with CD had decided not to travel alone any longer or even stopped traveling completely.

C) Meeting family and friends: Technology can facilitate socializing but not solve the stress of socializing

Socializing with family and friends was mainly related to positive perceptions. It was, however, also challenging. Technology was not part of the socialization, but was important as an enabler, in terms of e.g. reminding of coming appointments. According to the participants, challenges related to socializing mostly appeared outside home, at family and friends’ homes, and to some extent at home in terms of attending to birthdays or being on time for appointments, for example, deciding when to leave home. Being on time was described as a prerequisite for many social activities, and being late for an appointment or not being at home when visitors were expected could be perceived as very stressful by persons with CD. In order to avoid such mistakes, support from technology in the form of calendars or reminders in smartphones was used, and some also had access to different types of time aids; for example a PDA with reminders or features that enabled them to stay aware of the time. Family members were often supportive in managing appointments, which enabled persons with CD to meet with friends and acquaintances or attend meetings.

Not surprisingly, socializing with others was described as very meaningful, important, and appreciated. However, socializing was also closely related to stress and embarrassment, and the stress was mainly related to having conversations in the social setting at hand. This meant having to manage complex information in terms of finding subjects to talk about, describing thoughts and remembering earlier events or situations, saying socially appropriate things, finding the right words, and dealing with the intensity in loudness and tempo in conversations. One person with CD said: *“I think it’s tough (mm), I think it’s tough. HERE, it works really well, because I’ve met you and you know who we are and so on. But among others, I think it’s really tough. I feel so excluded. I’m afraid of talking, I might get stuck on some words, or say something wrong (sigh) so that’s what I think …”.*

Meeting people was described as very demanding and tiresome. It often implied the risk of embarrassment and also feelings of shame due to the consequences of the cognitive deficits, and even small family gatherings could be perceived as hardly worth the effort. This perception was also related to the feelings of not being able to contribute to the group, and some told of becoming increasingly quiet when meeting people, while others withdrew and avoided social gatherings altogether. For some, the only solution was to exclude themselves from valuable social relations. In some cases, however, it was not the persons’ choice to withdraw, instead, it was reported that friends had withdrawn after the cognitive deficits had been noticeable. In one case, a participant (wife of a person with CD) described how her husband left his hearing aid at home when visiting his children and thereby could blame his bad hearing when he could not follow the conversation, rather than tell his family about his cognitive deficits. No examples were found in which technology was used to support socialization – just reminders and time aids. In contrast, the findings show that the need of appearing as no different from others and saving face could make persons with CD hide such assistive technologies for cognitive support, e.g. time aids in PDAs.

D) Engaging with culture and media: Technology impacts the use, but enjoying the actual content could be the biggest challenge

When engaging with culture and media, the challenging activities appeared mostly at home and often related to technology. The challenges could involve attending to specific points in time for TV programs, as well as managing the interaction with the technology, e.g. the TV, the DVD, and the variety of remote controls. Not being able to handle technologies that were used ubiquitously by people in one’s surroundings, such as smartphones or tablets, could convey feelings of irritation, sadness, disappointment, and loss. In one specific case, a participant described how her husband with CD became very disappointed when he wanted to learn to use a tablet for watching fun film clips but could not manage it, although he had been very competent in using similar things before. She said he told her: *“Well, I’m not allowed to drive any longer and I can’t manage this either”.* The tablet was thereafter not used, since it made him so sad.

However, the challenge in engaging with culture was not always related to the use of technology, but rather to the requirements of the valued activity itself, such as the need to concentrate to be able to enjoy different types of media, or to remember the plot in films and books. Cultural activities and their value had previously been taken for granted, and not being able to take part in them any longer conveyed feelings of sadness, irritation, or anger. This was described by a participant with CD:

*“… when I worked, I always read the paper in the morning and that was like (zzip), and it was done. Now, I have to sit half the day if I were to read the whole (morning paper). And then, when I have come to the last page, I can’t remember anyway what I’ve read. (Laughter). It doesn’t stick in the same way any longer … I would never be able to do that anymore. Those things make me sad, actually. I think that’s a bit sad.”*

The main strategy described by the participants was to adapt activities with the purpose of maintaining them in some sense. For example, some persons with CD decided to read less complex books, re-read books that they had read before, or solve crosswords intended for children. In some cases, family members had to take over the handling of the media technology, and when the computer or the DVD became too complicated, it had in some cases been phased out without any feelings of loss.

The technology could also be viewed as supportive to enable enjoying culture, if it was possible to use it in other ways than before, for example, using computers or tablets for enjoyable or stimulating tasks such as reading, playing games, or solving crossword puzzles. Especially media in the form of computers or tablets could provide means for training cognition, and this was generally perceived as positive**.** As an example, some people with CD played a popular computer game for enjoyment but also for training their cognitive skills.

E) Doing everyday chores: Technology can threaten home safety and independence yet afford reliable reminders

Everyday chores that were described as challenging were by their nature performed frequently, and they were primarily performed at home, except for shopping. Most technology linked to the challenges in this area of concern was offline, which differed from most other areas.

When performing everyday chores at home, in terms of cooking and baking, there was a high risk of forgetting to turn off household equipment, which made technology part of the challenge. However, a more frequently mentioned challenge in this area was using recipes in terms of keeping the procedures in mind when cooking, which led to repeated reading. Cooking and baking was hence time-consuming, and not being able to prepare the food as timely and organized as before was related to sadness, irritation, and anger. One participant with CD said: *“Yes, I get irritated by it, because I had been used to having my hands full. I have done that kind of work. And now, everything goes so slowly for me, and I think that’s tiresome. It almost makes me feel a little depressed instead. I don’t get angry, instead I think I am so slow because it takes such a long time when I’m about to prepare food, which has been easy before. It took me a whole day to prepare a lasagna! Well, It would have taken an hour, because it is a bit complicated, but, but … (sigh). It took me a whole day to prepare that darned lasagna and that’s insane!”* Stories were told of when persons with CD wrote down instructions for their own use or started preparing less complicated or well-known meals to reduce the need to read recipes. Unsurprisingly, some cohabiting persons with CD had given up cooking. Technology was not mentioned as means to support the persons with CD when cooking and baking. Other challenging everyday chores performed at home included remembering small everyday tasks related to health, such as taking medicine, brushing teeth or eating. Contradictory to the challenges perceived while cooking, using technology as a support in health management was not uncommon. For example, persons with CD inserted reminders in a mobile phone, smartphone or a PDA with the intention to avoid forgetting to take medicine or brushing teeth.

Outside the apartment, in the common laundry room in the housing complex, a relatively new challenging chore had emerged; handling the online booking system for the public laundry room, a common facility in Swedish housing. For this activity, there was a need to quickly navigate on a screen to set one’s preferred date and time for the laundry before being logged out and also to understand unfamiliar symbols. In one reported case, the high demands from the technology, i.e. the booking system, had become a severe hindrance; doing the laundry was no longer an activity performed independently.

Outside of the home, the only challenge mentioned in relation to everyday chores, apart from payment, was to remember what items to buy when grocery shopping. Mobile phones and smartphones were very useful for calling a partner in order to be reminded about what to buy in the store. This exemplifies how the combination of using the technology and having support from a person at a distance to get correct information was a supportive solution.

### The involvement of technology on challenges in everyday activities

The involvement of technology in the five areas of concern was very contrasting. Technology as hindering and evoking stress, and therefore challenging, was particularly found in areas of concern linked to Managing personal finances, Engaging with culture and media and Doing everyday chores. When the challenges appeared at home, they were linked to managing finances on the computer and handling household equipment or media. When challenges linked to technology appeared outside home, they occurred when making payments or withdrawing money in stores, other public places or when using public transport.

Interestingly, there were also examples of technology being a part of the solution in the same three areas of concern as those where technology was hindering and evoking stress. Solutions were often linked to using mobile phone functions as reminders or checking online status reports for expenses and invoices. Not surprisingly, reminders set in the mobile phone were highly used and could meet some challenges, thereby enhancing control when Managing personal finances, Meeting family and friends and Doing everyday chores. However, the support from the technology could be too limited; for example, in the area Getting around, the GPS function could fail to support the completion of an activity due to its complexity. It was evident, however, that the technology was neither a part of the problem nor the solution for challenges when the expectation was to take in and enjoy the content in a film or to have a social conversation. In those cases, the more common approach to meet the challenge was to stop doing the activity.

## Discussion

In this study, five areas of concern in everyday life were identified as offering challenges to persons with CD: A) Managing personal finances, B) Getting around C) Meeting family and friends, D) Engaging with culture and media and, E) Doing everyday chores. Not surprisingly, the challenging activities identified in these areas were to a great extent similar to the challenging activities that persons with CD wanted to continue mastering according to a previous literature study [19], thus empirically verifying former findings. As the challenging activities were found to be of great and existentially profound importance to people with CD [19], enabling these people to come to terms with the identified challenges in the equivalent areas of concern found in the present study may be decisive for success when striving to make a positive impact on everyday life for people with CD. The results of the present study add to previous findings by identifying the contrasting role of technology both as part of the challenge as well as the solution within these five areas of concern, taking the environments in which the challenge occur into account. In addition, a broad variety of approaches to meet the challenges utilized by persons with CD were identified. These approaches span from simplifications (e.g. use of paper forms/paper calendars, choosing less complex activities) to use of smart technology (e.g. GPS). The overview presented in Tables 2, 3, 4, 5 and 6 shows that technology being part of the problem was as common as technology being part of the solution, with variations between the areas of concern. The insights into the role of technology in activities that persons with CD value yet find challenging provided by the concrete examples in the findings will hopefully clarify and suggest new ideas on how and for what purpose to design technological support. For example, when the goal is to design support for the identified challenges, knowledge about the part that technologies and environments might play in the challenge can be used in the development process together with knowledge of how persons with CD have met the challenge. How technology can be involved in challenging activities in different environments will be further elaborated in this discussion.

The area of concern in which technology was found to be most problematic was Managing personal finances. Considering the importance of financial activities for everyday life, as well as the common difficulties in financial ability shown early after onset of CD that previous research has underscored (e.g. [30]), there is a need to emphasize the urgency of this problem. It was evident in our findings that one technological obstacle in this area was the user interfaces of digital financial services. These had a crucial influence on the outcome when managing personal finances, regardless of whether the activity was conducted at home or in public places. The findings show how persons with CD, as we all do, meet a variety of technological payment or withdrawal systems, including Internet banking and on-line shopping websites, or payment terminals at the local shop, either at the cashier or at the automated check-out station, and, further, at vending machines and ATMs. Considering the difficulties commonly related to memory and new learning in persons with CD (for example, MCI [9, 14]), the variation and lack of congruence between user interfaces may be an important obstacle to the possibility of persons with CD to use these services. The need not only for web access but also increased usability of the web has been stressed as necessary for the full and equal enjoyment of web content by people with cognitive disabilities [31]. Further, the fear of financial abuse also appeared in our findings as well as in others’ [32], and this must not be neglected. Being victims of financial crime is not uncommon among older people with cognitive deficits [33] and the feeling of vulnerability might negatively influence the wish to continue engaging in certain everyday activities and to be involved in society, resulting in withdrawal.

In our findings, there were many examples of technology being a part of the solution when meeting challenges in different areas of concern, but the solutions were in most cases limited to services linked to the mobile phone or specific websites. Reminders in the smart phone could support a person with CD to initiate important activities, keep appointments or remember medicine intake, and personal account information on the bank website was regularly checked to confirm whether bills were paid. In these particular cases, it is important to point out that even if the technology did not support the activity per se (e.g. taking medicine or paying bills), it showed to be supportive enough to enable some persons to initiate the activity and to maintain control. It should, however, not be taken for granted that because a person can initiate a challenging activity, they can perform and complete it. Moreover, only addressing a part of the challenge might not make the expected positive change for a person with CD. This was evident in the area of concern Getting around, where technology offered potential support, but at the same time created challenges. It has been stated previously that persons with CD often stop going on outings and traveling due to the demands linked to it [5, 35] and that GPS applications could support continued outdoor activities [23], thereby supporting wellness, enjoyment [36] and even social encounters [37]. Being able of finding one’s way comprised many stressful challenges and the supportive technology – the GPS service – was, on the one hand, described by participants as highly needed for persons with CD for location and directions and consequently enabling valued activities outside home. On the other hand, however, using the GPS on a smartphone was described as too complicated to handle. Thus, the GPS technology was not perceived as being able to fully support the person in activities linked to orientation. This illustrates how one group with obvious needs for GPS support, i.e., persons with CD with well-documented orientation deficits [34], is at risk of being excluded from the use of this vital service, and consequently from valuable activities outside home, due to an interface and functional demands that do not meet their specific needs. It is further important to take into account that, according to these findings, challenges related to handling household equipment at home was mostly linked to leaving the home safely. Providing technology that ensures that the home is secure when leaving it has shown to decrease perceived stress when persons with CD perform activities outside home [23]. Further development of services in this area would be beneficial.

In the area of concern Meeting family and friends, the findings showed that - apart from reminders - technology was neither part of the problem nor the solution when meeting challenges in socializing. One approach to meet challenges related to socializing was instead to withdraw from social gatherings completely since the gains did not outweigh the challenges. Withdrawal from social contact has been reported also in previous studies [38, 39] and could be a consequence of embarrassment and stress [40, 41]. However, it is well known that social engagement is crucial for wellbeing [42] and that loneliness has a negative influence on cognitive capacities and may speed up the rate of cognitive decline [43, 44]. As previously mentioned, the participants in this study did not describe technology as supportive when socializing. Other studies [45] have shown that e.g. smart phones have been beneficial for socializing, for example, for sharing photos and more frequent contacts. The importance of significant others as support in technology use for socializing has been underscored [45]. However, the goal of using technology might differ between significant others and persons with CD, and if a conflict arises, the person with CD is more likely to have the weakest voice [46]. Even if technology for social engagement as a means to decrease the speed of cognitive decline is important, it should not overshadow the role of technology as means to maintain valued social contacts; they are of crucial value in their own right. Yet, it is important to acknowledge that the challenges identified in our findings often were related to the perceived quality of conversations, a much more complex matter than just being able to stay in contact.

One goal of this study was to identify approaches that were used by persons with CD to meet challenges, because paying attention to individuals’ self-initiated approaches may reveal their resources and be useful for guiding the development and provision of support [38]. However, such approaches may also pose new problems to the person with CD. Even if individual approaches to manage challenges are necessary, it is plausible that changes in the environment offer more possibilities. According to the Person-Environment-Occupation Model [24], the “*environment is considered to be more amenable to change than the person”* (p. 17) and changing aspects of the environment can support a compatible fit and thereby increase the person’s performance of an activity. When aiming at supporting persons with CD to remain engaged in activities at home and in society, it is important not to neglect the technological environment as one target for change, since most complex challenges were linked to this environment both at home and outside home in our findings. Since technology was shown to be involved in many challenges that occur outside home, often in public spaces and shops where individual support might be less applicable, there are reasons to explore how technologies in public space could be designed to diminish known key challenges and thereby support persons with CD. This is also in line with the WHO initiative for Age-friendly environments [47], which intends to provide accessible public spaces and transportation that enable independence and participation in community life. An age-friendly environment provides services and support to compensate for the loss of function so that people can continue to do valued activities. To accomplish this, it would be beneficial if decision makers would request as well as facilitate that technological services in public space are designed to enable persons with CD to access and use them. Not considering the functional requirements of technology on persons with CD when designing services for e.g. buying tickets or withdrawing money will hinder their continued engagement in society [31]. The technological solutions that persons with CD would benefit from would very likely be supportive also to other people, in line with the guidelines of inclusive design, defined as “the design of mainstream products and/or services that are accessible to, and usable by, as many people as reasonably possible ... without the need for special adaptation or specialized design”. [48].

### Methodological considerations

It is important to bear in mind that the areas of concern are inductively developed representations of the areas where challenges are experienced and how persons with CD interact with technologies in everyday life, according to the participants in this study. This means that research involving other participants might result in other or additional representations. Most participants in the two focus groups representing voluntary health organisations were females, which might have led to a gender bias. Moreover, the focus group discussions were conducted in 2013. The fast development in technology has probably made especially social activities on the Internet more common. Technological support has also become more accessible and usable. On the other hand, we do not know if people with CD have adopted new technologies and services. Consequently, it is possible that new technologies have become part of new problems, just as well as they might be part of new solutions. Anyway, our findings should be interpreted with the presented limitations in mind.

## Conclusion

This study focuses on the involvement of technology in those areas of concern where persons with CD encounter challenges. Findings showed that the involvement of technology in everyday activities was very contrasting, both in public spaces and within the home. It could be hindering and evoke stress or, in contrast, bring about feelings of control – that is, being a part of the solution. The involvement of technology was especially obvious in challenges linked to managing personal finances, which is a crucial necessity in many everyday activities. In contrast, technology was neither a part of the problem nor the solution for challenges when socializing with family and friends, suggesting that technology itself is not the solution to socialization problems, but rather one medium to facilitate staying in touch. Findings imply that technology used for getting around and managing finances, often outside home, would particularly benefit from being further developed in order to be more supportive; i.e. accessible and usable. In order to make a positive change for many people who face challenges in getting around and managing finances, the ideas of inclusive design seem fruitful, as does the WHO initiative of age-friendly societies. As two areas representing public rather than domestic life; getting around and managing finances, came to the fore in our findings, this implies redistributing the balance between designing for the individual and redesigning the environment, especially the public environment and the Internet.

## Additional file

Additional file 1: Topics guide for focus group discussions. This file presents the interview/topics guide for the focus group discussions. (DOCX 30 kb)

## Abbreviations

AAL: Ambient Assisted Living, or Active and Assisted Living
CD: (Mild) cognitive deficits
GPS: Global positioning system
MCI: Mild Cognitive Impairment
PDA: Personal digital assistant
