# Supplementary material for: The contrasting role of technology as both supportive and hindering in the everyday lives of people with mild cognitive deficits: a focus group study
Source: BMC Geriatr. 2018 Aug 17;18:185. doi: 10.1186/s12877-018-0879-z (PMC6098644; doi:10.1186/s12877-018-0879-z)
Supplement: Supplementary file 1 — Topics guide for focus group discussions. This file presents the interview/topics guide for the focus group discussions. (DOCX 30 kb) [file 12877_2018_879_MOESM1_ESM.docx]

**Topics guide for focus group discussions**

Questions are phrased slightly differently to the different types of participants (professionals/researchers and members of volunteer health organisations). When needed, triggers are provided based upon examples from the ongoing discussion, from earlier focus groups, from the literature or from what is commonly known. Follow-up questions are continuously used, for example: When? Where? Can this be done in other places? With whom? What is the impact e.g. from culture, laws and regulations?

*Start:*

General information is given about the research and how data are treated to ensure confidentiality, about the focus group approach and the aim of the discussion.

*Introduction:*

Participants are invited to speak about how they come into contact with (people with) cognitive problems/issues.

*Difficulties experienced:*

Participants are invited to share and discuss their own examples of situations in which they/persons with cognitive impairment may experience difficulties, and *what* in those situations had become more difficult.

*Priorities:*

Participants are invited to share and discuss their own examples of

- situations that often are experienced/described as difficult by persons with cognitive impairment,
- difficulties/situations that are especially important to manage for/according to persons with cognitive impairment, and why these situations are important to manage
- situations that may be more upsetting than others when one cannot manage them for/according to persons with cognitive impairment, and why these may be more upsetting
- how difficult tasks/situations can be managed in alternative ways or avoided

*Environments where problems are encountered and potential consequences:*

Participants are invited to share and discuss their own examples of how different types of environments such as physical, social, infrastructure (telecommunication and internet communication), laws, rules and regulations, attitudes, cultural and time aspects, may interfere in situations such as those exemplified and discussed as difficult and/or especially important to manage.

*Readiness for use of technology and assistive devices:*

Participants are invited to share and discuss their own examples of

- reactions and opinions (own and others’) on using everyday technology, and of technological solutions to cognitive problems
- ethical aspects of technology use

*Visions for the future:*

Participants are invited to share and discuss their views on

- important developments or steps that would help people with cognitive impairment manage their daily lives
- technical devices or technical solutions that do not yet exist (“imagine if”)

*Validation of the literature review:*

The facilitator gives a short

- description of what the earlier literature review showed; then inviting to a discussion on how those findings agree/disagree with participants’ experiences.
- description of issues that had been identified in the previous focus groups; then inviting to a discussion on how those findings agree/disagree with participants’ experiences.
- summary of the interview’s key elements; then asking if there is anything else participants would like to add.

*Closing the focus group*

Information is given about how the material will be used in the project. The facilitator thanks participants for their time and participation and asks if they agree to be contacted again later if needed for clarifications.
